# Blood Pressure Variability as a Risk Factor for Cardiovascular Disease: Which Antihypertensive Agents Are More Effective?

**DOI:** 10.3390/jcm12196167

**Published:** 2023-09-24

**Authors:** Alejandro de la Sierra

**Affiliations:** Hospital Mutua Terrassa, University of Barcelona, 08221-Terrassa, Spain; adelasierra@mutuaterrassa.cat

**Keywords:** blood pressure variability, antihypertensive treatment, cardiovascular disease, cardiovascular risk, ambulatory blood pressure monitoring

## Abstract

Blood pressure oscillations during different time scales, known as blood pressure variability (BPV), have become a focus of growing scientific interest. BPV can be measured at long-term (seasonal variability or visit-to-visit), at mid-term (differences in consecutive days or weeks) or at short-term (day-night differences or changes induced by other daily activities and conditions). An increased BPV, either at long, mid or short-term is associated with a poor cardiovascular prognosis independently of the amount of blood pressure elevation. There is scarce evidence on the effect of different antihypertensive treatments on BPV, but some observational and interventional studies suggest that calcium channel blockers in general, and particularly amlodipine, either in monotherapy or combined with renin-angiotensin system blockers, can reduce BPV more efficiently than other antihypertensive drugs or combinations. Nevertheless, there are several aspects of the relationship between BPV, antihypertensive treatment, and clinical outcomes that are still unknown, and more work should be performed before considering BPV as a therapeutical target in clinical practice.

## 1. Introduction

High blood pressure (BP) is one of the most powerful determinants of cardiovascular risk [1]. BP estimates, obtained punctually in the clinic or through measurements in different hours, days or weeks, are associated with the risk of cardiovascular events and mortality [2]. However, BP is not a static component, and fluctuates due to a large number of internal and external influences. Blood pressure variability (BPV) is defined as the BP variation over different time scales, ranging from beat-to-beat to years [3].

The importance of BPV is that such fluctuations are related with the development of organ damage, cardiovascular events and mortality, independently of the absolute degree of BP elevation. Such circumstances have created a great interest in the understanding of mechanisms responsible for BPV, the different types of variability and the methods of assessment, as well as possible therapeutical interventions modifying several aspects of BPV.

## 2. Types of Blood Pressure Variability, Physiological Regulation, and Methods of Assessment

The types of BPV, their physiological regulation, and methods of assessment depend on the time scale contemplated. It ranges from very short periods of time, such as beat-to-beat or even intra-beat variability, short-term variability, usually defined as variations occurring in a 24 h period of time, day-to-day, week-to-week, or long-term variability, including variations occurring among visits, in different seasons of the year, or even through several years [3,4].

The physiological regulation of BP variations, as well as its derangements, are complex and poorly understood. They constitute a mixture of cardiovascular regulatory mechanisms, as well as behavioral and environmental factors [4]. In addition, several treatments, and both cardiovascular drugs and non-cardiovascular drugs, influence BPV. Among the intrinsic mechanisms regulating BPV, baroreflex activity and arterial stiffness are possibly the most important. Short-term variability is highly dependent on the circadian rhythm of activity and sleep and, during sleep, is particularly affected by sleep disturbances. The adherence to antihypertensive treatment and the duration of action of different antihypertensive drugs clearly influence day-to-day, week-to-week, as well as visit-to-visit variability. Changes in weather and outdoor temperature are the main influencers of seasonal variability.

The assessment of very-short-term BPV requires continuous monitoring. This can be achieved by intra-arterial recording, or with the use of some non-invasive devices. Its use is usually restricted to monitor patients in intensive care units, emergencies or operation rooms. Continuous ambulatory non-invasive devices using finger plethysmography were developed several years ago, but were impractical for its use in ambulatory patients [5,6]. Short-term BPV is usually assessed by Ambulatory Blood Pressure Monitoring (ABPM) with oscillometric validated cuff devices, measuring BP at repeated intervals, usually from 15 to 30 min [7]. Home Blood Pressure Monitoring (HBPM) is the method of choice for assessing mid-term BPV, such as day-to-day or week-to-week changes. It is usually recommended to proceed to a 7-day period of measurement, twice per day, three repeated measurements each [8]. The results of this schedule are very close to daytime BP obtained through ABPM. Finally, office BP is usually the method of assessment of visit-to-visit variability or seasonal variability. It is necessary to strictly adhere to a protocol of measurement, which should be always the same. Minor deviations can cause important differences in BP, which are not necessarily patient-dependent [3].

## 3. Blood Pressure Variability Indices

The standard deviation (SD) and the coefficient of variation (the ratio between standard deviation and absolute BP value) are the most commonly used indices for all types of BPV. Variability independent of the mean has been also proposed for the assessment of visit-to-visit variability, and it is calculated by dividing SD by the mean powered to a value obtained by non-linear regression analysis of population values [9].

Several other indices are also commonly used for short-term BPV. Among them, the most important are weighted SD and average real variability (AVR). The first is obtained by the average of daytime and nighttime SD weighted for the duration of each period [10]. Its main advantage over the classic 24 h SD is that is not affected by the amount of nocturnal BP decline. ARV is obtained by calculating the average of the differences (in absolute value) between consecutive measures [11]. It better reflects within subject variability, although is more affected by poor quality of the data.

## 4. Prognostic Value of Blood Pressure Variability

The first and most consistent data regarding the prognostic influence of BPV refers to long-term or visit-to-visit variability. A post hoc analysis of four large clinical trials, including a large cohort of patients with a history of transient ischemic attacks (UK-TIA Aspirin Trial) and a large population of patients with hypertension and added risk factors (ASCOT-BPLA) demonstrated that intervisit BP variability and peak BP values were strong predictors of stroke independently of mean SBP and, albeit to a lesser extent, equally associated with coronary risk [12]. Since then, these findings have been reproduced in both cohort studies [13] and post hoc analyses of other clinical trials, especially in subjects at high risk for the development of cardiovascular diseases [14,15,16]. In contrast, other studies in hypertensive patients without added risk or in the general population have not been able to determine a significant contribution of long-term variability between visits to cardiovascular morbidity and mortality, which would suggest that the association between BP variability between visits and cardiovascular outcomes could be significantly influenced by the individual’s baseline cardiovascular risk level. These discrepancies were also revealed in a meta-analysis of 23 studies in which, although the variability between visits was associated with the development of coronary and cerebrovascular events, as well as with the number of total and cardiovascular deaths, the degree of association in all cases was modest and did not exceed a 20% increase in risk [17].

Regarding mid-term BPV and cardiovascular prognosis, two population studies have confirmed the prognostic value of BP variability in the medium term. In the Ohasama study [18], increased variability values in home SBP, measured over a total of 26 days, was associated with a higher composite risk of cardiac death and stroke. Moreover, in the HOME-BP study in Finland, carried out in a cohort of adults from the general population, increased variability in systolic and diastolic BP measurements for seven consecutive days was associated with an increased risk of cardiovascular events after almost 8 years of follow-up, which remained significant even after adjusting for age and mean HT levels, thus supporting the additional value of home BP variability in predicting cardiovascular prognosis [19]. This has been recently confirmed using the multinational IDHOCO database [20]. In contrast to these results, a study with 12-year follow-up in a Belgian population did not show any predictive value for BP variability when adjusting for mean BP values [21]. However, in this latter study, BPV was estimated from only two home visits.

With regard to short-term BPV, as measured through ABPM, the relationship has been established both with the standard deviation estimators, night pressure fall and with the morning surge. An increase in the standard deviation both during the day [22] and at night [23] has been related to a worse cardiovascular prognosis in prospective studies. This relationship is even more evident when the aforementioned estimators of weighted standard deviation [24] or average real variability [25] are calculated.

The nocturnal fall in BP also has an important impact on cardiovascular prognosis. The first studies already suggested that a lack of nocturnal decrease in BP was associated with a worse cardiovascular prognosis [26]. The data from the Spanish ABPM Registry [27] have revealed that the prevalence of these “deleterious” patterns is very high, and that they are close to 50% in untreated patients and exceed this figure in those who are under treatment. Advanced age, female sex, obesity, diabetes, and a history of previous cardiovascular disease are associated with inadequate decline in both treated and untreated patients. In the former, the number of drugs also intervenes in a greater probability of presenting a “nondipper” or “riser” pattern.

The main problem in assessing the prognostic value of circadian pattern alterations is the association of both nocturnal BP fall with nocturnal BP levels, a very well known factor for a higher risk of mortality and cardiovascular events [28].

Not only the nocturnal decrease in BP, but also the increase that occurs upon awakening may be important from the prognostic point of view. Thus, in the Japanese population or those of other Asian countries, an excessive increase in the morning has been associated with a greater risk of events, especially cerebral vascular accidents of hemorrhagic etiology [29]. However, the actual prognostic significance of this morning rise remains a matter of debate, given the significant positive correlation between the degree of morning BP rise (a potentially deleterious phenomenon) and the degree of nighttime BP fall (a potentially protective phenomenon). In addition, the morning increase that occurs in Caucasian populations appears to be clearly less than that in Asian populations. In the former, when both elements are analyzed in the same group, the importance of the lack of nocturnal decrease seems to be greater than the excess of morning increase [30].

## 5. Blood Pressure Variability and Antihypertensive Treatment

Meta-analyses of clinical trials in hypertension with different classes of antihypertensive drugs have strongly supported that mean BP reduction is essential to achieve cardiovascular protection [31]. Moreover, it has been suggested that the reduction in long-term variability provided by some drug classes may confer additional benefits in addition to lowering mean BP levels. In support of this concept, a meta-analysis comparing the calcium antagonist amlodipine against other antihypertensives suggested a favorable impact of the former on blood pressure variability between visits [32]. In the ASCOT study, there was also a parallelism between the greater impact of amlodipine compared to atenolol on long-term variability and protection against stroke [9], although it should be recognized that other advantages of amlodipine with respect to atenolol, such as a greater impact on central BP reduction, were also present in this study [33]. This favorable effect of amlodipine on visit-to-visit variability was also observed in the SPRINT study [34], and it could possibly be related to the long half-life of this drug, of about 30 h. It has also been hypothesized that certain drug combinations might be more effective than others in reducing long-term variability. In this sense, the combination of an angiotensin receptor antagonist with a calcium antagonist was capable to promote a greater reduction in blood pressure variability between visits compared to the same receptor antagonist combined with a diuretic, regardless of the reductions in mean BP levels [35].

With respect to mid-term BPV, studies on the effect of antihypertensive treatment are few and quite inconsistent. Only one comparative study evaluating the effects of two types of antihypertensive combinations found that a combination of an angiotensin receptor antagonist with a calcium antagonist was more effective in reducing home SBP variability than the combination of the same receptor antagonist with a thiazide diuretic [36]. Finally, a non-randomized analysis of a population of diabetic subjects who received different classes of antihypertensive drugs found lower morning BP variability values in the subjects who received calcium antagonists, compared to those treated with angiotensin converting enzyme (ACE) inhibitors or angiotensin-receptor blockers (ARB) [37].

With respect to short-term BPV, again studies have shown a beneficial effect of calcium channel blockers, particularly amlodipine. The X-CELLENT study [38] showed a greater reduction in short-term variability in patients treated with amlodipine, or the diuretic indapamide in comparison to the angiotensin receptor antagonist candesartan. Another study in treated hypertensive patients showed that subjects who received calcium antagonists or diuretics, alone or associated with other groups, had significantly lower SBP standard deviations compared with those who received angiotensin-converting enzyme inhibitors, receptor antagonists or beta-blockers [39].

In the Spanish ABPM Registry, we looked at short-term BPV in a very large number of patients under different types of antihypertensive therapies, including monotherapies and different combinations [40]. A total of 38,188 patients were included. BPV indices, including daytime and nighttime SD, weighted SD and average real variability increased as the number of antihypertensive drugs increased, being statistically significant when compared the group receiving three or more drugs with those on monotherapy.

In this latter group of patients treated with monotherapy, the comparison among the major drug classes revealed lower BPV indices in those treated with calcium channel blockers or diuretics, in comparison with beta blockers, ACE inhibitors or ARB. When looking at the different compounds inside each therapeutic class, there were no differences in BPV among diuretics (hydrochlorothiazide, chlorthalidone or indapamide), while in the calcium channel blocker group, amlodipine was associated with lower values of BPV, in comparison to other dihydropyridines, diltiazem or verapamil. Finally, this favorable effect of calcium channel blockers, particularly amlodipine, was also observed in patients treated with a two-drug or a three-drug combination, with those including a calcium channel blocker being associated with lower values of BPV indices.

It has recently been suggested that new non-pharmacological treatments for resistant AHT, such as renal denervation, could have a beneficial effect on short-term variability. In this regard, in a clinical trial comparing treatment with spironolactone versus renal sympathetic denervation, the latter procedure had a greater effect on the short-term variability of diastolic pressure, measured by the 24 h weighted standard deviation or by the average real variability [41]. A recent meta-analysis has also concluded that renal denervation favorably affects short-term BPV in patients with resistant hypertension [42].

## 6. Clinical Significance, Practical Recommendations and Future Directions

There is considerable theoretical evidence that abnormalities in BPV are associated with the cardiovascular prognosis. However, BPV is a general term including several possible abnormalities, which in fact may have different pathogenetic mechanisms. It is difficult to admit that differences in BP among long-term visits or seasonal variations have any relation with day–night changes or other changes in shorter periods of time. They are all considered BPV, but mechanisms responsible are obviously different. Moreover, it is still a matter of debate if increased BPV is a true risk factor influencing the prognosis or merely a marker of other alterations. In addition, all the evidence regarding the impact of therapeutical maneuvers on BPV are based on post hoc analyses of clinical trials, in which the primary objective was the cardiovascular prevention through the achievement of an absolute BP reduction.

Considering these limitations, it can be hypothesized that the impact of different antihypertensive treatments, in reducing BPV in addition to their effect on absolute BP, might be an advantage in terms of protection. Results from clinical observations suggest that long-acting calcium channel blockers have an advantage over other types of drugs. However, if this advantage is related to the mechanism of action or to their pharmacokinetic properties is still unknown. New drugs under development include some possible changes in the way antihypertensive treatment is currently administered. The possibility of using drugs administered once per month or even once or twice per year will open different perspective in the assessment and the role of BPV.

Not only possible changes in antihypertensive treatment, but future changes in BP measurement and monitoring will also impact in the study of BPV. The growing use of wearable BP devices represents a unique opportunity, and may change the way BP is measured in the future. They have obvious advantages, as the possibility of monitoring BP very frequently, almost continuously, without interfering individual activities and without tolerability issues is clearly promising. These aspects are of particular relevance in the evaluation of BPV, and can serve for future studies. However, as it has been recently stated [43], the accuracy of such wearable devices has not been unequivocally demonstrated, and this is an obvious previous requirement before they can be ready for clinical use.

## 7. Conclusions

It is possible that the different classes of antihypertensives may have a different impact on blood pressure variability, at short, mid or long terms. If this could be translated to a greater cardiovascular protection, independently or added to the decrease in mean BP values will need specific clinical trials to be assessed. It is also possible that not only the antihypertensive drug class, through its mechanism of action, but also the pharmacokinetics of each compound would impact BPV, with drugs with longer duration of action reducing more efficiently BPV.
